# Call to action: A literature review of Chagas disease risk in California 1916–2018

**DOI:** 10.1371/journal.pntd.0009035

**Published:** 2021-02-25

**Authors:** Alba Valdez-Tah, Carlos N. Ibarra-Cerdeña

**Affiliations:** 1 Department of Anthropology, University of California–Irvine, City of Irvine, California, United States of America; 2 Departamento de Ecología Humana, Centro de Investigaciones y Estudios Avanzados del IPN (Cinvestav) Unidad Mérida. Mérida, Yucatán, México; Universidade Federal de Minas Gerais, BRAZIL

Human Chagas disease (CD), caused by the *Trypanosoma cruzi* infection, is considered nonendemic in the United States of America [https://www.cdc.gov/parasites/chagas/gen_info/detailed.html#intro; consulted March 29, 2020], assuming that this chronic affection’s epidemiology is different from that of Latin American countries. The attention is mostly focused on blood bank screening, organ donation, and vertical (mother to child) transmission [1]. This approach can overlook the potential for autochthonous transmission of *T*. *cruzi*, particularly in the light of a recent acknowledgment about the role that vectorial transmission may have on the CD in the USA. A low awareness among physicians about CD risk besides its migratory origin can contribute to an existing underreporting rate, thus masking the accurate estimation of CD burden in the USA [2].

Cases of autochthonous infection in the USA are not accurately tracked since it is only mandatory to report it in seven states, i.e., Arizona, Arkansas, Louisiana, Mississippi, Tennessee, Texas, and Massachusetts [3]. However, as it occurs in Latin America, where the index of suspicion is much higher, acute *T*. *cruzi* infections in immunocompetent individuals can pass undiagnosed and undetected among residents [4]. In the USA, 238,091 cases are estimated as of 2012 [5]. This analysis highlighted the role of Latin immigrants in the CD burden, with California being the state with the highest estimated number of cases.

Although imported CD cases are undoubtedly a matter of concern, there are also environmental conditions leading to autochthonous infections [6]. New encounters between humans and triatomine bugs are often associated with the destruction of or invasion into vertebrate hosts’ habitats, compromised housing structures, or both. Disruption of host burrows provokes the bugs to seek new refugees, and their attraction to artificial light often leads them to nearby human dwellings [7]. Multiple publications have highlighted the southern USA as an area where autochthonous infection occurs, mainly because of the high proportion of impoverished residents living in substandard housing infested by triatomines [7–10].

It is essential to improve our knowledge of *T*. *cruzi* infection risk components because there are parasite reservoirs among wildlife species and vectors living in contact with humans [11]. This is especially found in less-studied regions such as California, where the epidemiological, parasitological, and entomological patterns of *T*. *cruzi* transmission might resemble those of other endemic areas. Surveillance of infection prevalence among local populations of Triatominae is critical for accurate assessment of the public health risk. We attempt to contribute to this with an extensive historical literature review of records of autochthonous *T*. *cruzi* and human-related data for CD in California. This method of review provides a contemporary account of this topic’s breadth of knowledge. There has been no recent review of this theme at the state level to the best of our knowledge, as has already been conducted in some other states, such as Texas [12].

We summarized a historical account of triatomines reports, infection with *T*. *cruzi* in triatomines, mammals and humans (CD), and human-triatomine interaction for California counties. We found 62 articles published between 1916 and 2018 encompassing 35 countries (Table 1), mostly from the greater Los Angeles area, metropolitan San Diego, Sierra Nevada’s foothills, and the Morongo Basin (Fig 1A and 1B).

**Fig 1:**
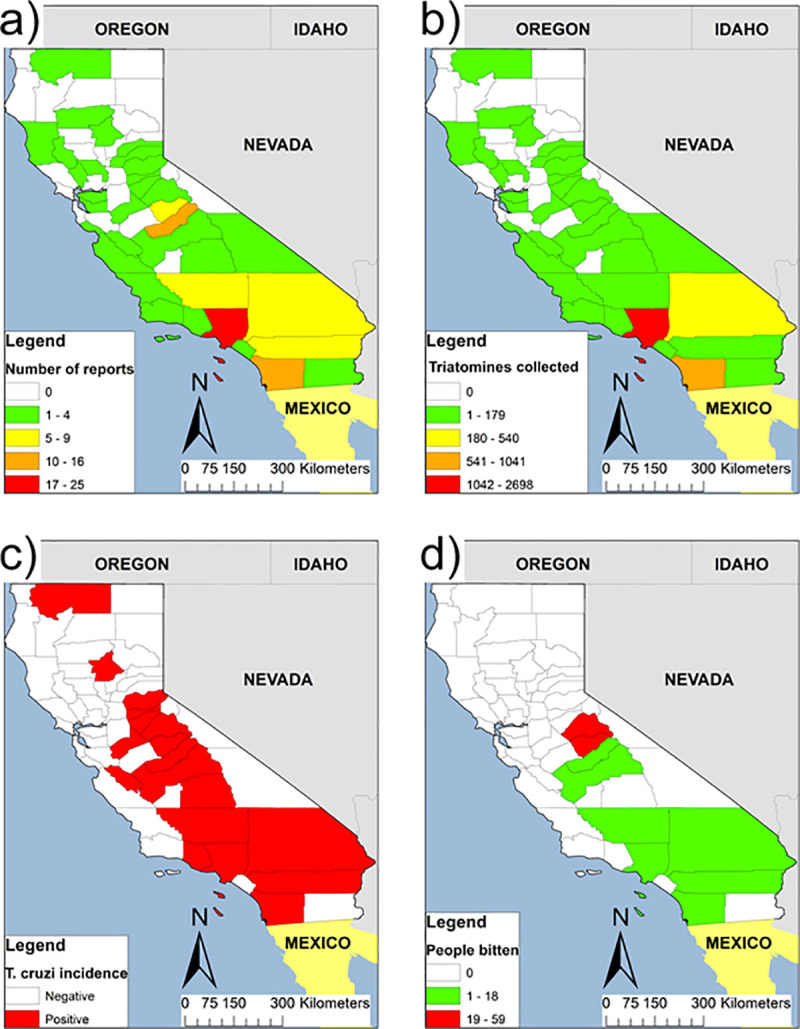
(A) County-level distribution of the literature reviewed of autochthonous *Trypanosoma cruzi* transmission and Chagas disease in California 1916–2017; (B) Published reports of county-level abundance distribution of Triatomine species; (C) Published reports of county-level incidence of natural infection of *T*. *cruzi* among triatomines and mammals; and (D) Published reports of the distribution of human exposure (manifested as bites) to triatomines, reported at the county level. Counties in white are more likely regions with no reports. Geographic boundaries were obtained from the 2016 TIGER/Line shapefiles prepared by the United States Census Bureau. TIGER/Line Shapefiles (machine-readable data files): Current County and Equivalent National Shapefile. 2016.

**Table 1:** Temporal distribution of publications, triatomines collected, and first county reports of autochthonous *Trypanosoma cruzi* infection.

Decade	% Publications	# Triatomine collected	Counties of first report
**1930**	5	652	San Diego, Los Angeles, Inyo, Kern
**1940**	7	289	Fresno, Riverside, Amador, Madera, Stanislaous, Tulare
**1950**	12	731	San Bernandino
**1960**	14	2,427	Ventura, Butte, Mariposa, Mendocino, Monterey, Napa, Contra Costa, El Dorado, Lake, Yolo, Alameda, Orange, Nevada, Placer, Santa Barbara, Santa Cruz, Siskiyou, Tehama, San Luis Obispo
**1970**	6	353	Imperial, San Benito
**1980**	7	110	Tuolumne
**1990**	1	0	--
**2000**	3	191	Calaveras
**2010**	7	137	--

Between 1916 and 1930, *T*. *cruzi* was reported for the first time in the USA in San Diego County. The Los Angeles and San Diego counties account for up to 1,000 and 2,500 records, respectively. The *T*. *cruzi* infection prevalence was estimated for 34 of 56 localities in 13 counties (Fig 1C), ranging from 9% to 100% among triatomines and 0.62% to 100% among six wild mammals and dogs (Tables 2 and 3). The most abundant triatomine species in California is *Triatoma protracta* (98% of the 4,951 records), followed by *Triatoma rubida* and *Paratriatoma hirsuta*.

**Table 2:** Prevalence of *T*. *cruzi* infection among triatomines per county.

County	No. of specimens collected	No. of specimens analyzed	No. (%) Positive for *T*. *cruzi* infection	References
**Kern**	1	1	1 (100)	[13]
**Tulare**	2	2	2 (100)	[14,15]
**Calaveras**	44	44	29 (65.9)	[16,17,18]
**Madera**	125	77	39 (50.6)	[14,19,20]
**Butte**	4	4	2 (50)	[13]
**Los Angeles**	2,005	1,679	576 (34.3)	[13,17,19,20,21,22,23,25,26]
**San Diego**	1,027	477	157 (32.0)	[6,21,27]
**Fresno**	4	4	1 (25)	[27]
**San Benito**	9	9	2 (22.2)	[28]
**Riverside**	18	18	4 (22.2)	[29]
**Stanislaus**	6	6	1 (16.7)	[14]
**San Bernardino**	398	355	54 (15.21)	[30]

**Table 3:** Natural *T*. *cruzi* infection among mammals, according to the county.

Species	County	Total no. Tested	No. (%) Positive for *T*. *cruzi* infection	Assay type (sample or specific assay)	Site of collection	Reference
**Skunk (*Mephitis mephitis*)**	Los Angeles	1	1 (100)	Serology and histology	Peri-urban (Griffith Park)	[31]
**Wood-rat *Neotoma* spp.Big-eared woodrat**	Los Angeles	99	9 (9)	Xenodiagnosis and blood smear	Wild, Peri-urban	[32] [28] [15]
	San Diego	228	2 (0.88)	Blood smear (from the heart)	Wild	[33] [34]
	Calaveras	49	7 (14.3)	PCR assays	Wild (within private property) Microhabitat on private property	[6]
**Gilbert white-footed mouse (*Peromyscus truei gilberti*)**	Madera	484	3 (0.62)	Xenodiagnosis, and blood smear	Wild	[35] [36]
**Pinon mouse (*Peromyscus truei montipinoris*)**	Los Angeles	228	12 (5.3)	Xenodiagnosis	Peri-urban	[15]
**Dog (*Canis familiaris*)**	Tuolumne	17	6 (35.3)	CF Titers and Culture (blood) (1982) 4 seropositive dogs: Antibodies CF Titers and IIF (1984)	Domestic	[37]

The first report of domestic incidence was recorded in the 1940s. In 38.8% of the publications reporting collection site for triatomines (19/49), at least one was collected inside human habitation. Domestic triatomine incidence was associated with dispersion from the vicinity of residences in semirural, canyon, or mountain-foothill areas (involved removal of mammal hosts and triatomine nest); during the monsoon, houselights are attractive to triatomines, especially while mating and/or seeking blood-meals.

Twenty-two publications reported 164 triatomine bites and ensuing anaphylactic reactions among residents of 11 California counties (Fig 1D), 80% of them taking place inside dwellings. Tuolumne and Los Angeles counties reported the largest number of cases. At least four patients with Romaña signs have been reported since 1964.

In publications around the 1950s, the bulk of domestic triatomines reports and human exposure came from residents’ testimonies, from Kern, Riverside, Fresno, and San Diego counties, and from descriptions of clinical cases (S1 Text). Although in California, CD is not mandatory to report. In 2008 and 2010, the Californian Vector-Borne Department Section recorded reports of vector inside homes and bites. A different perspective of this interaction are the 24 distinct common names in English, Spanish, and a Native American language for the insect found across the literature. This might suggest it’s a more common interaction than previously thought. This has been indicated by ethno-entomology studies and reports among indigenous peoples within the CD endemic areas (Table 4) [38].

**Table 4:** Common names for triatomines in California reported in literature.

Triatomine name	Reference
Kissing bug	[39]
Cone-nose bug, China bedbug, Crossbug	[40]
Bellows bugs, blood-suckers, suckers, Walapai Tigers (Wali’s), Cactus bugs, Bedbugs, Big bedbugs, Chinese bedbug	[41]
Mountain chint bug	[24]
Western blood-sucking cone-nose, Monitor bug	[42]
Diamond-back bug, steel-pin bug, chinche	[43]
Assassin bug, Cannibal bug, Pirate bug, Mexican bedbug, Giant bedbug	[44]
Cone-nose beetle	[45]
Western cone-nose bug	[12]
Vampire bug	[46]

Research on human-triatomine interaction in California involved two surveys conducted 50 years apart. The State Department of Health conducted a study in 1962 in Mariposa and Tuolumne counties that recorded more than 101 bite cases. In 2012, Los Angeles’ residents reported bites resembling the swelling (called Chagoma) produced by triatomines, and rural residents in Santa Barbara were found with immunoglobulin G (IgG) against the *T*. *protracta* saliva. Along with this, human blood-meals have been found in free-roaming triatomines [47].

In 1982, the first autochthonous human case of CD was reported for California in Tuolumne county: a 50-year-old woman from Lake Don Pedro [48]. Thereafter a broad serological survey, the only type in the states’ history, was conducted among three groups of residents, revealing antibodies in 2.5% to 0.7% of the population. The second case was reported in Simi Valley in 2016 and discovered through a routine screening test in blood-donation, a 19-year-old white male, locally born and raised, with no acknowledged bite but who had presumably been exposed during outdoor activities [49].

This review of the autochthonous transmission of *T*. *cruzi* in California is the first one in more than 50 years. A temporal analysis of the publications shows an early scientific effort to compare some endemic areas; 80% of the articles were published before the early 1980s (Table 1). Amid the current debate about autochthonous transmission in the Southern USA, evidence of an ongoing domestic cycle and *T*. *cruzi* human infection has been found [7,9]. Our review shows evidence that the current assessment of public health risk among California residents is mostly based on outdated and is nonpublicly acknowledged.

Regarding the low number of human CD cases in California, our literature review shed light on a set of circumstances that have difficulted the report of official autochthonous cases. First, human-triatomine interaction was assessed in terms of risk of anaphylaxis—common in the Southwest—but not for *T*. *cruzi* transmission. Only one bite case was followed up by a screen test. A triatomine bite is nonreactive among 85% to 95% of the cases, reducing the chances of seeking medical care and suspicion of *T*. *cruzi* infection [50]. Secondly, there is no routine screening program at the primary care level in California, and mandatory blood-donor screening is not adequate for capturing *T*. *cruzi* infection among both Latino immigrants and residents [1]. Current efforts in this sense target Latino immigrants. Therefore, as potential autochthonous cases, individuals might not meet risk criteria about the country of birth, ethnicity, and a history of travel to an endemic country. The lack of specifically assessed locally acquired infection and large-scale serological surveys might have failed to detect autochthonous cases. Lastly, physicians might be unaware of the local risk and fail to encourage patients to get tested [2]. Health providers have underestimated CD cases because they believe it to be “a South American problem,” leading to misdiagnosis [51]. In patients’ cases, they may decline to seek healthcare if feeling healthy during the CD indeterminate phase (nonsymptomatic), when having limited access to healthcare, i.e., among the low-income and homeless individuals, or when choosing to avoid a perceived stigma [52]. In the USA, mainstream stereotypes are that CD is a “tropical exotic” disease prevalent among so-called “illegal aliens” and it is associated with poverty.

Compared to what occurs in Latin America, a generally higher housing standard in the USA has been a main contributor for the low incidence of reports of domestic triatomines and human CD [47]. However, both the historical domestic incidence and recent data documented here suggest that residences might harbor vector colonies [53]. While triatomine oviposition, nymphal development, and *T*. *cruzi* infection may occur outdoors, we believe that *T*. *cruzi* transmission in California might have some similarities with Texas, and Mexican endemic triatomines are not domestic but disperse from surrounding areas. Human-triatomine interaction also occurs outdoors due to the free-roaming vector that feeds on human hosts given the opportunity [7,10]. In California, outdoor activities within forests and natural parks are widely popular, for instance, during 2018, a total of 269,055 hunting licenses were granted, and campsite attendance reached 7,265,525 overnight stays.

Socioenvironmental changes of the last five decades in California have not been followed by research on their effects on human-triatomine interaction and *T*. *cruzi* transmission. Loss of habitat and human settlements (and increasing numbers of pets) have led to an increasing encroachment upon the natural habitat of triatomines and their mammal hosts. This might raise the chances of triatomine domestic incidence due to their flexibility in habitat and host requirements, including dogs and other mammals (rodents, opossums, raccoons, etc.). Examples of *Triatoma sanguisuga* in Louisiana came after the disruption of hurricane Katrina; there was a change of infestations in human dwellings [53].

County-level maps of reports only partially reflect the *T*. *cruzi* distribution in California. This is due to the scarcity/absence of records in some areas and early discovery of the association triatomine-woodrat that might be biased to the field research. An ecological niche modeling approach of the geographic distribution of vector species indicates favorable host habitat in many unsurveyed regions of California [54].

Our review in California highlights that a critical and accurate assessment of the public health risk for *T*. *cruzi* and CD needs research/intervention on the:

1. Spatial modeling of pattern occurrences of triatomine and *T*. *cruzi* infection for further robust analysis on habitat suitability and risk due to socioenvironmental changes;
2. Dynamics and mechanisms of transmission: spatial variation of triatomine activity, infection levels and their reliance on humans-blood, dynamics of dispersal flights and free-roaming triatomines feeding habits;
3. Identification of sylvatic, synanthropic, and domestic (especially dogs) animal blood-sources and determination of infection variability;
4. Identification of the *T*. *cruzi* circulating strains and an analysis of the parasite’s genetic variation;
5. Large-scale systematic programs of active surveillance, rigorous population-based data collection, and comprehensive assessment to determine infection prevalence and morbidity to identify factors that might inform policy and program-driven actions, especially among the vulnerable (impoverished residents living in substandard housing);
6. Improvement of local knowledge local about epidemiology and ecology and the criteria for public health promotion to raise awareness among residents, physicians, and clinicians;
7. Active screening of heart failure patients, hunters/campers, and pregnant women; and
8. Continuing medical education programs on diagnosis and treatment among physicians.

## Supporting information

S1 Text Domestic and peri-domestic reports of human bites and human exposure to vectors. (DOCX)
