# Supplementary material for: Call to action: A literature review of Chagas disease risk in California 1916–2018
Source: PLoS Negl Trop Dis. 2021 Feb 25;15(2):e0009035. doi: 10.1371/journal.pntd.0009035 (PMC7906329; doi:10.1371/journal.pntd.0009035)
Supplement: S1 Text — (DOCX) [file pntd.0009035.s001.docx]

**Suplement 1. Evidence table containing all published reports of human bites, and human exposure in domestic and peridomestic space in California.**

| Year | County | Issue | Literature extracts | Reference |
| --- | --- | --- | --- | --- |
| 1938 | Kern | Bite/ Exposure | 1. “Nothing is known concerning the habits of this species beyond a report from J. W. Vernon that the specimens from Mojave had attacked man” (105). | [1] |
| 1942 | Fresno | Bite  Infestation | 2. Resident testimony: “This bug bit me on the hand last night. […] They affect my wife differently. She will not feel the bite usually but the next day will have a bad headache […] I have known people in the neighborhood to become unconscious from the bites but thought it might be hysterics” (43-44).  *Triatoma specimen sent to the author by residents | [2] |
| 1942 | Fresno  Riverside | Bite  Infestation | 3. Resident testimony: “This neighbor was bitten two years ago and again last year by one of these insects […] The neighbors’ little girls was also bitten and in each case the effect was the same […] These insects come in the night and especially seem to know where the bedrooms are for they stay on the ceiling then drop on the bed later […] Then my neighbor was bitten by this insect and we found a number of them a short time after the sheep left […] We seldom see one in daylight, but they have the uncanny habit of remaining on the ceiling directly over a bed, then when anyone lies on that bed the bug drops down” (63).  4. […] Several people have been bitten lately and made ill” (64).  5. “He (a resident) reported that as within the past few years he saw as many as 5 *Triatoma* (probably *protracta*) or as he called them, “kissing bugs,” o the screen at night” (64).  6. Resident testimony: “The first attack was July 4^th^… I was bitten several times and finally caught one of the bugs in the act” (64).  7. Resident testimony: “I am sending you a bug today that bit me twice last night. Once on the hip and once on the forearm” (64).  8. “On July 5, 1941, concerning a neighbor from whose residence the writer found one bug infected out of four received, Mr. Reynolds writes as follows: ‘He says that he finds then around his bed nearly every morning’” (64).  9. “The woman who lines in the stone cabin-found a whole nest of them [triatomines]. She said there were about twenty, mostly small ones, in an old glove” (64).  10. *Triatoma specimens sent to the author by residents | [3] |
| 1950 | San Diego | Bite  Infestation | 11. Resident testimony: “Kissing bugs get into our bed. When my husband is bitten he puffs up around the eyes and his tongue gets thick. His throat swells up and he itches from head to foot. What kind of poison do they pump into him?” (149).  12. *A triatomino found inside bed | [4] |
| 1950 | Fresno  Stanislaus | Infestation | 13. “He reports two seen in his canyon home during the summer of 1948 and one in a neighbor’s house, whereas only three specimens were observed in the summer of 1949” (98).  14. “Four [triatomines] came from a lumber pile near a house in the fall of 1948, and eight came from a residence in the fall of 1948, and spring and summer of 1949” (99).  15. *Triatoma specimens sent to the author by residents. | [5] |
| 1950 | Los Angeles  Madera | Bite  Infestation | 16. Resident testimony: “‘Last summer I had a bite which caused a condition similar to hives (urticarial or angioneurotic edema)’” (71).  FALTA  17. Resident testimony: “Positive proof or the cause is lacking, but my wife saved for me the bug which she found on the wall above the baby’s bed soon after the symptoms were first noted” (71).  18. Mr. Z’ was bitten four or five times on the back by something about ?:00 A.M., after returning from work at midnight and retiring. Soon afterward Mrs. ‘Z’ found a *Triatoma protacta* under his pillow. The bites were not at first noticeable” (71).  19. “Mrs. ‘Z’ and two teen age daughters report that they all have been bitten, but suffer only local itching and swelling” (72).  20. *Triatoma specimens sent to the author by residents. | [6] |
| 1951 | Madera | Infestation  Bite  Infection | 21. “Plate 35 shows the floor plan of this house with pertinent features indicated to show bug location at time of capture […] Two concentrate of Triatoma are noticeable, one in the bedroom and one on Porch C” (106).  22. “The bugs gain access in the summer months to the living space from the sides, especially around doors, or the under surface of the house. There were many white footed mice (*Peromyscus maniculatus gambelii*) and several Pacific tree frogs (*Hyla regilla*) living under the house” (108).  23. “Forty-four *Triatoma protracta* (19 males, 25 females) were collected between July 1^st^ and August 18^th^, 1950 from this house. Fecal droppings of 43 were examined for blood parasites and 19 or 44% were naturally infected with *Trypanosoma cruzi* Chagas” (108).  24. “The dining room served as sleeping quarters for the two children. Two male bugs were taken, one on the floor and the other on the moulding” (109).  25. “The bedroom yielded at least 14 specimens. Two male *Triatoma* fed upon the occupants and were found the following morning at the top end of the mattress and in the crack between mattress and box springs at the head of the bed” (110).  26. “As is noted in Plate 1, six specimens (1 male, 5 female) from the bedroom were infected with *Trypanosoma cruzi* including one male which fed on the sleeping occupants of the room.”  27. “An additional 17 specimens were reported or collected form six other houses by… [names of residents]…” (110). | [7] |
| 1952 | Madera | Infestation  Infection | 28. “*Triatoma protracta* (Uhler), Wstern Conenose. This bug not only feeds on the wood rat but also enters homes and feeds on man. In 41’ from homes, Lowell Adams forwarded 8 (5 males, 3 females) bugs of which 5 were infected. In 49’ Lisle Green and Keneth Wagnon sent 17 (12males, 5 females) bugs for examination and 12 were infected with trypanosomes” (52).  29. “In ’50, 56 ( 22males, 34 females) bugs were obtained from homes and 5 others reported destroyed, the bulk being taken in one dwelling whose occupants served as food for at least 2 bugs (Wood, Fifty- five of these bugs were examined for trypanosomes and 25 were found infected. In ’51 and ’52, 18 (12 males, 6 female) bugs were examined from homes with 5 Of 9 infected in ’51 and 6 of 9 infected in ’52. 29. One 5th nymph from the horse barn was negative in of 104 bugs taken from homes, 98 were examined and 58 were found naturally infected with *Trypanosoma cruzi* Chagas” (52).  30. “The western conenose has been taken from beds and observed to crawl across the ceiling and drop to the bed below. At a ranch on Hildreth Road, *Triatoma* was observed to fly from a corner of the room to a seated person’s lap” (52). | [8] |
| 1953 | Los Angeles | Bite/  Exposure  Infection  Infestation | “Additional annoyance was reported to the writer from Alpine and in the California Farmer (Anonymous, 1950), both for San Diego County, and from Thermal in Riverside County (Verbal communication). One severe reaction to *Triatoma* bite in Griffith Park was reported although at least 8 others living permanently in the same locality showed no reaction” (105).  “Recently, many more naturally infected bugs were collected and more bug contacts with man were noted as a result of home invasions by these insects” (105).  “Severe reaction to feeding of *Triatoma protracta* including nausea, palpitation of the heart, breathlessness and severe itching have been noted in one case of 9 adults (6) and children (3) exposed to the feeding of this bug in Griffith Park. Although others have evidently served as host for blood meals of *Triatoma*, no bite or systemic reactions were noted” (105).  “…while 33 were collected from temporarily or permanently occupied human habitations […] only 17 were examined from homes, one infected *Triatoma* was found from a permanent residence and one from temporary quarters” (105).  “They appeared chiefly in the early evening and were found inside the buildings, in the folds of blankets and in one instance under a pillow in the morning. Rafferty estimated 25 home-invading adults *Triatoma* were found from June 20^th^ to September 1ts” (106-107). | [9] |
| 1958 | Los Angeles | Infestation | “The first adult Triatoma found in human habitations during 1957 was taken at the Boy’s Camp on July 2^nd^ and the last at the Girl’s Camp on September 28^th^ with the heaviest invasions occurring during July and August. Two hundred thirty-four conenose bugs were taken in human habitations…” (39).  “Speciments of Triatoma were collected from buildings, camp grounds, residences and wood rat houses” (39).  “Three bugs collected from campers or their beds were foun engorged with blood indicating possible recent feeding on man” (40).  “Near Area 60, 1 male and 4 female Triatoma were found in a home bordering the Park by Ruth S. Stein. All were negative for trypanosomes” (42).  “The discovered of naturally infected conenose bugs in camper’s luggage suggest possible disemination of infected bugs to Los Angeles homes” (45). | [10] |
| 1960 | Los Angeles | Infestation  Infection | “Conenose bugs were found from June 24^th^ through September 13^th^ at the Boys’ Camp […] Bugs were found from July 2^nd^ through September 18^th^ at the Girl’s campt” (50).  “Additional interesting locations of live bug captures were inside a shoe, a suitcase, a washing machine, and between cardboard stripps in the craft room. As noted in Table 1, most bugs were capture inside the buildings” (50).  “The 1957 infection rate of bugs from the Boy’s Camp area was 38.8% as compared with 40.8% for 1948 and 14.2% rate for the Girls’ Camp area. The 1958 infection rate for male bugs was 25% and for female bugs 45.9% at the Boys’ Camp and 30% and 8% respectively at the Girls’ Camp. This is especially significant from the Public Health point of viewpoint since it reveals the existance of continued human contacts with bugs having a high enough natural infection rate with *Trypanosoma cruzi* to be significant in contaminative transmission of Chagas’ disese” (51). | [11] |
| 1961 | Los Angeles | Infestation | “During the summer of 1960, 364 bugs were collected from the youth camps, 189 from the Boys’ Camp and 175 from the Girls’ Camp;…” (191).  “One case of a severe reaction to the feeding of *Triatoma*, on September 23, 1960, was reported to the senior author. The case ocurred in a home located near the boundary of Griffith Park at the bottom of a chaparrar covered slope. Upon investigation, 1 male *Triatoma* was found in a dresser drawer; 2 first instars that had recently fec were found on the bedroom rug; and 1 first instar that had not yet taken a blood meal was found in a cardboard box under the bed. Fecal deposits on the wall at the head of the bed, and in a box under the same bed indicated the feeding on Triatoma over a period of time” (191). | [12] |
| 1962 | Toulomne  Mariposa | Bites/ Exposure  Infestation | “During the past three years increasing numbers of complaints have been received by the Bureau of Vector Control from the Sierra Nevada footsills. In particular, Mr. Herbert Davis, Mariposa County Area Sanitarian, reported a large number of T. protracta bites and severe reactions among residents of the county. Dr. Norman Nichols, Mariposa County Health Officer, requested the assistance of the Bureau of Vector Control to assess the public health importance of this problem” (33).  “One hundred ten case histories of *Triatoma* bites were collected during 1961 from 71 females and 39 males. Most of these were from Mariposa and Toulumne counties. In every case the patient had been bitten on more than one ocassion […] Over 95% of these cases had collected the bug which had bitten them. A significant number of these bugs were submitted to the investigators or the Mariposa County Health Department. All of these specimens were identified as *T. protracta*. Persons interviwed during the study were shown a specimen of *T. protracta* which they confirmed as the same type of bug that had bit them” (34).  “All but one (109/110) were bitten within the home. Most persons found the engorded bugs in their beds. In residence with heavy infestations the bugs are apt to be found anywhere within the home. In homes subjected to periodic annoyance, the bugs were largely limited to the bedroom, living room and porch. Bugs frequently enter houses through openings resulting from shifts in the foundation (Wood, 1951). Other means of entry reported on the questionare were gaps around doors, windows, or the undersurface of the house, or on clothing or bedding which had been hanging outside and then carried into the house” (35).  “In addition to beds, bugs were often found in bedroom closets, especially in pockets of cloithin and in shoes. They were also found under rugs, behind drapes and pictures, and crawling around crevices and baseboards. Ocassionally they were seen on walls or crawling on the ceiling” (35). | [13] |
| 1963 | Mariposa | Bite | “Case 1. A 14-year’old girl was awakened at 3 a.m. with general itching and a few hives scattered over her body […] In most cases an engorged kissing bug can be found in or around the bed covers, if careful search is made” (267). | [14] |
| 1963 | Los Angeles | Bite  Infestation | “A few weeks ago, a very worried young mother brought in a 5 or 6-year-old son whose cheek had obviously been puncture […] She also brought in for identification a cone nose beetle which she declared had been the cause of the wound” (17).  “Shortly after moving in, his wife was ‘bitten’ by a cone nose beetle. The wound was again in the upper cheek. Shortly after receiving it she became quite seriously ill and was hospitalized for weeks […] A few days after her recovery and return home she attended a late afternoon social affair at a neighbor’s home and again was ‘bitten’ by a cone nose beetle […] Afterward, we found several dead cone nose beetle, and worse, they were found in her bedroom. In desperation we took off the inlet screen cover from the air duct system, and in this series of ducts we believe that we found the source” (17). | [15] |
| 1963 | Los Angeles | Bite  Infestation | “Shortly after this, she became nauseated, got up to go to the bathroom, almost fainted, fell back in bed, and called her husband. Her husband turned on the light, assited her, noted a bug on the wall behind the bed, and killed it. (The patient’s husband had served in the China-Indian-Burma Theater during World War II and stated that he ‘reflexly’ kills any bug that he notices)” (977).  “The patient was advised to search for the bug that had been killed during the night, and this was found and identified by the Department of Entomology at the Department of California, Los Angeles, as a *T. protracta*. […] The patient was residing in the upper Bennedict Canyon region of the Beverly Hills, Calif. At the time of the bite, the patient discussed her illness with neighbors. She stated that several others had had similar episodes” (978). | [16] |
| 1964 | Los Angeles | Infestation  Infection | “However, in 1960 when these nocturnal flights were occurring in Sepulveda Canyon, bearby residents were reporting *Triatoma* annoyance. Two males and 3 females were received from inside a Sherman Oaks home on July 24, 26, and September 29. One male and 1 female showed heavy infections with *Trypanosoma cruzi*. A Westwood resident collected 3 males and 6 female on July 18 and 21. Two males were positive for Chagas’ trypanosome; 1 bug was found inside the home. One male and 1 female *Triatoma* collected September 12 and October 4 from separate residences in Roscomare Canyon were heavily infected with *T. cruzi*” (775). | [17] |
| 1964 | Madera  San Diego | Infestation  Bite | “A chicken ranch on Hildreth Road, Madera Co., table 1, in the Sierra Nevada foothill country near O’Neals is apparently attractive to *Triatoma*. Inspection of a newly constructed residence revealed an unscreened fireplace vents as the site of entry for night-flying conenose bugs. The wife of the ranches reacted so violently to the feeding of *Triatoma* that she refused to occupy this location overnight. The presence of dogs for property protection furnished another strong attraction for conenose bugs to this well-built mountain home” (105).  “The Alpine, San Diego County, location represents a 12-year cooperative donation of interested housewives. Forty-two specimens were from one home, seven form homes immediately adjacent and two from within one mile of the others […] Recorded observations of 24 *Triatoma* by Mrs. E.A. Wilcox in her home reveal that 15 bugs were found in the kitchen and bathroom. Nine were taken from the kitchen sink and one from the bathtub during spells of hot weather. Other collecting sites were living room (3), bedrooms (3), upstairs landing (1), and back room where the dog slept (2) […] One bug was found hiding in a slipper and another in a boy’s sock. One bug flew in and landed near the refrigerator one summer evening” (105-106).  “Mrs. F. D. Fitzsimmons and neighbors reported at least 50 specimens destroyed up to July 21^st^ during the spring and summer of 1959. She reports that many times she observed bugs lying on their backs on the floor […] She observed an adult bug feeding on the paw of a sleeping cat on the davenport in the living room without disturbing the pet. She reports the first case in California, known to the authors of a *Triatoma* feeding on the face (eyelid) of man, with subsequent edema” (106).  “The Roscomare Canyon reached our laboratory as a result of special interest by local physicians in the presence of this home invading disease vector. It is most unusual for single samples that both bugs from inside these homes harbored heavy infections of Chagas’ trypanosome” (107). | [18] |
| 1965 | Los Angeles | Infestation | “Only 4 were successful in entering the screened quarters of the resident caretaker which is located in the flight path of the bugs to the lodge. These four bugs might have entered unnoticed on clothing or from repeated openning and closing of the screened door” (348). | [19] |
| 1966 |  | Infestion  Bite | “During the flight season 16 T. protracta were found within the residence. […] The actual collection dates by the occupants of the house are as follows: 17 June (dead specimen); 27 & 30 July; 9, 27, 28, & 31 August (2 bugs); 5, 9, 21 & 24 September; and 7, 11, & 18 October (2 bugs)” (89).  “Three bugs had taken a very small recent blood meal. A fourth bug collected in the house had fed to repletion on one of the residents during the night of 21 September” (90). | [20] |
| 1967 | Los Angeles | Infestation | “Subsequent collections by the senior author, his students, and the camp caretaker, Arthur L. Gladwill, have revealed a remarkably active population of conenose bugs preying on man and other large mammals during the normal dispersal flight in the park camp area 29 & 30 at 118° 17' W. Long, and 34° 08' N. Lat” (538).  “During summer dispersal flights, triatomes were handpicked from the inside or outside surfaces of human habitations within 1.6 km of wood rat houses” (538).  “During the 1965 summer at the Boys’ Camp, additional observations were made for the 67 triatomines from the caretaker’s quarters and the ground floor foundation of the former director’s home at 229.2m elevation” (541).  “The caretaker's home is two stories, the upper being the living quarters and the lower serving as a garage and storage area (fig. 5). Seventeen *Triatoma* were collected by Gladwill during 1965. Of 8 picked up inside the home, 3 ^ and *30-* harbored *Trypanosoma* *cruzi* and *20-* were negative. Of 7 found in the garage and storage area, 4°- were positive, *2$* negative and l<y was not examined. For 2 triatomes found on the outside steps leading to the living quarters, 1°- was positive and 1 ^ was negative” (541).  “A number of factors have played a part in the recovery of triatomes (Table 1) at the Camp Director's home. Allergic sensitivity to the feeding of *Triatoma* has alerted all occupants to a high degree of awareness for the presence of this unwelcome nocturnal visitor leading to an early evening search and capture of actively wandering insects. Additional heavy applications of insecticidal sprays have flushed out the triatomes from hiding places […] The presence of two large dogs, bedded outside the home, first at the Boys' Camp and later at the Girls' Camp have aided the recovery of *Triatoma* due to their attractiveness (verbal communications from kennel owners) as blood sources for this bug” (544). | [21] |
| 1975 | Mariposa | Bite | “Broddrick had been bitten several times by the bug, suffering severe reactions and had been heard to say, ‘Some day that dam bug is going to kill me. Friday it did” (347). | [22] |
| 1975 | Siskiyou  Los Angeles | Infestation  Infection | “1 positive (female) from a home in Yreka, Siskiyou Co., 12 July 1967, forwarded from the State Department of Agriculture by R. Hawthorne; 1 positive (female) from the living room couch in a home in the Hollywood Hills, Hollywood, Los Angeles Co., 20 August 1967, E. DeRover Jr….” (167).  “From 1964 through 1970, 28 *T. p. protracta* were reported to me from homes in Beverly Hills (Benedict Canyon), Los Angeles County. Microscopic examination of the feces of 3 (females) and 11 (masculinos) revealed 1 (female) positive for *T. cruzi* form inside a home 7 September 1968 and another (female) positive from an outside screen 8 July 1969” (167).  “During the summer of 1965, A. L. Gladwill, caretaker at the Griffith Park Boys’ Camp in Los Angeles, California, collected 13 *T. p. protracta* (6 male, 7 female) from the vicinity of his living quarters […] one female alive 30 August 1965 from inside the home revelaed 1 trypo/ and 8 epi-mastigotes when examined 310 days after capture!” (167). | [23] |
| 1975 | San Diego | Bites/ Exposure | “Since 1935 I have had letters, phone calls and personal interviews from many persons ‘bitten’, i.e., fed upon by the blood-sucking western conenose bug, *Triatoma protracta protracta* (Uhler)” (19).  “ Since 1963 I have used a questionnaire to be filled in by the bite victims. I have received written reports from 142 persons bitten by triatomes […] My reports indicate that for most areas where *T. p. protracta* feeds on man, 5 to 15% of the human residents may react in some degree to the foreign proteins infected by salivary fluids of this blood-feeding insect” (20).  “This bug, collected 16 February 1956, was known to have produced a ‘severe reaction’ involving generalized edema, closure of both eyes with swollen eyelids, and temporary paralysis from a bite in the arm pit of an adult female who lived on a ranch near Escondido, California” (20).  “I investigated one man, age 60, a severe reactor who acquired his sensitivity to *Triatoma* saliva in 11 years, probably involving 3 to 10 feedings per year (Wood, 1950)” (21). | [24] |
| 1978 | Ventura | Infestation | “Evidence of home invasions in Thousand Oaks included 1 negative male, 1 infected female, and 1 infected male from Woldwood Parkside Trac residences. Three adults not examined nor include in the above tally were taken in another Parkside home on 23 August 1977, 2 being found in the bedroom” (130). | [25] |
| 1982 | San Diego | Bite | “The patient’s first known encounter with *Triatoma protracta* occurred during the summer of 1977. Reactions at the site of the bite began with intense local pruritus and prominent edema with local pyrexia. […] At least 6 bites were sustained over a period of 3 months…” (249).  “An event involving bites on the eyelid and neck resulted in the eyelid being swollen shut for 5 days and cervical lymphadenopathy for some weeks afterward… […] In August 1978 the patient received a bite during the early morning hours, causing her to wake with a ‘strange’ feeling” (249). | [26] |
| 1984 | Los Angeles | Bites  Exposure | “Patient I is a 48-year-old man with a history of mild allergic rhinitis but otherwise no allergic problems. In November of 1980, he awoke from sleep with generalized pruritus and shortness of breath. On attempting to arise from the bed, he experienced postural hypotension and collapsed. His blood pressure was 60 mm Hg when paramedics arrived and administered epinephrine. The patient responded promptly. No etiology for the anaphylactic episode was determined. Similar events occurred in Aug. and Sept. of 1981. However, on one of these occasions, the patient was able to identify an insect bite, and subsequently the insect was found in the bedding. The insect was brought to UCLA (University of California Los Angeles) and identified as *T. protracta*” (370).  “Patient 2 is a 32-yr-old man with no previous history of allergic problems. On Aug. 5, 198 1, he experienced generalized urticaria that progressed to hypotension that he thought started after observing an insect bite. The patient was taken to a local emergency room and brought the insect with him. The patient responded well to epinephrine. The insect was disregarded as a cockroach. The patient was told it had nothing to do with his symptoms. Approximately 8 hr later that evening, the patient had a recurrence of symptoms while sleeping and again required epinepluine administration. Three days later, the patient again experienced generalized urticaria and hypotension while sleeping and was referred to UCLA. After the earlier history was obtained, the patient was asked to bring in the insect that he originally suspected. It was identified at UCLA as *T. protracta*” (370). | [27] |
| 1985 | Tuolumne | Human infection  Infestation  Bite | “*Triatoma protracta* (a vector of *Trypanosoma cruzi*) infected with *T. cruzi* were found near the patient's home […] A serosurvey of three groups of California residents revealed antibody to *T. cruzi* by complement fixation in six of 237 (2.5 per cent) individuals living near the patient and in 12 of 1,706 (0.7 per cent) individuals living in a community 20 miles northeast of the patient's home, but in only one of 637 (0.2percent) blood donors from the San Francisco Bay area” (366).  “The patient's house had no evidence of chronic infestation with *Triatoma* or rodents, but a live, uninfected *Triatoma* *protracta* was found in the patient's bathroom. […] Two uninfected *T*. *protracta* were found in two of seven neighboring houses” (367).  “Human Serosurvey. The results of our human serosurvey are summarized in Table 2. Six individuals from Lake Don Pedro had positive CF titers (>8). Their ages ranged from 18 to 73 years, and they had lived at Lake Don Pedro for one to five years. One had traveled to northern Mexico six months before, but none had traveled to southern Mexico or Central or South America. Two had received blood transfusions, and one recalled being bitten by a *Triatoma*. All six of the seropositive persons owned dogs; however, 186 (80.5 per cent) of the 231 seronegative individuals also owned dogs. None of the seropositive persons had a history of a prolonged fever” (367). | [28] |
| 1999 | Amador  Mendocino  San Francisco Bay Area | Infestation  Bites/ Exposure | “*Triatoma protracta* nymphs and adults were collected mainly from the stick houses of woodrats and secondarily from human domiciles in Amador or Mendocino counties” (851).  “At the CHR, 9 (10.6%) of the 85 study subjects reported exposure to this bug (Lane RS, unpublished data), and therefore the 20% prevalence of AASA to *T. protracta* appears reasonable because many people bitten nocturnally would be unaware of their exposure unless they subsequently developed an allergic reaction. Similarly, 6.7% of residents inhabiting a community in a coastal chaparral-oak woodland area in southern California were found to be at risk for developing serious immediate hypersensitivity reactions to the bites of *T. protracta*” (855).  “The 10% prevalence of antibodies to *T. protracta* among residents of the San Francisco Bay region seems somewhat high. Although the woodrat host of *T. protracta* is present in parklands throughout this region, this rodent is absent in metropolitan areas where much of the human population resides” (856). | [29] |
| 2010 | San Diego  Los Angeles | Infestation | “Specimens were collected using light traps (ultraviolet and mercury vapor) in Escondido (San Diego County; 33°12¢44.9994²N, 117°5′36.9954²W, elevation = 405 meters) and Glendora (Los Angeles County; 34°9¢59.364²N, 117°50¢18.204²W, elevation = 391 meters), California. The sites are located at a suburban residence (Escondido) or less than 1 km from a suburban residence (Glendora). A total of 161 specimens (Escondido = 139 and Glendora = 22) were collected over an eight-week period (June–September 2008)” (1020). | [30] |
| 2010 | San Diego | Bite  Infestation | “Patient 3: anaphylaxis. A 46-year-old female resident of the foothills of rural San Diego County had been bitten by kissing bugs over a 7-year period, and the reaction was itching at the site of the bite. In September 2008, she was bitten once again, awoke scratching her left leg, and discovered that her face was red. She became too weak to walk, diaphoretic, and short of breath. Emergency medical technician services took her to a hospital, where her anaphylaxis was diagnosed and treated successfully. An adult male *T. protracta* insect (Figure 1) was discovered in her home on her bed comforter (Table 2). The patient’s daughter had been bitten as well and had a swollen tongue. She consulted an infectious diseases physician about the incident and was told that she need not worry about Chagas disease, because it was a ‘South American problem’” (1631). | [31] |
| 2012 | San Diego | Bites/ Exposure  Infection | “Five of the 13 bugs (38%) had positive test results for human blood (Table 1); *T. rubida* bugs were significantly more likely than *T. protracta* bugs to have fed on humans (χ29.24; p<0.01). *T. rubida* bugs had also fed on dogs and *T. protracta* bugs on woodrats (*Neotoma spp.*), chickens, dogs, and pigs. *T. cruzi* infection was found in 5/9 *T. protracta* and 0/4 *T. rubida* bugs. No insect that had fed on humans was infected with *T. cruzi* trypanosomes” (646).  “Around Escondido, we found *T. protracta* bugs fed on humans, woodrats, and domestic chickens, according to the cytB assay. This assay amplified only vector DNA from 4/7 insects, which could mean it had been a long time since the last blood meal and thus the DNA was highly degraded. We detected only 1 blood meal source in the other 3 insects from California but found 2 woodrat sequences in a single insect. Both *T. rubida* bugs collected in California had fed only on humans; 2 unique human sequences from 2 clones from 1 insect suggest it had fed on 2 humans” (648). | [32] |
| 2012 | Los Angeles  San Bernandino | Exposure  Bites  Infestation | “In the exposed areas 20% (95% confidence interval [CI]: 10–31%) interviewees knew about the presence of *Triatominae* in the neighborhood with a much higher awareness in the more rural desert area as opposed to the suburban community approaching the foothills (n = 41 [35%] versus n = 5 [5%]) (Table 2). When presented with the pinned specimens or insect images, 19% (95% CI: 11– 27%) participants reported to have ever seen *Triatominae* in the neighborhood and 8% (95% CI: 1–14%) reported to have seen them in the house during the month before the interview. As expected, kissing bugs were most frequently sighted between dusk and midnight (n = 11 [65%]), followed by sightings around dusks (n = 10 [59%]), and between midnight and dawn (n = 9 [53%]). Only 2 (12%) persons reported sightings between noon and dusk, and 1 (6%) person at noon. No sightings were reported between dawn and noon.”  “The number of *Triatominae* seen in the house during the month before the interview was below ten with two exceptions. One interviewee reported seeing 20 and another 100 specimens. Concordant with the nightly feeding habits and painless bites of these insects [4], only 7% (95% CI: 2–12) persons reported to have ever been bitten by *Triatominae*.”  “Exposure to *Triatominae* was corroborated for 10 [5% (95% CI:1−8%)] study participants in the exposed areas, who collected and sent us at total of 12 specimens that we identified as *T. protracta* (7 [3%] of participants with at least one specimen ) or as *P. hirsuta* (3 [1%] persons with one or more specimens). All insects except one were collected inside homes. Most of the insects were found within a few months after the survey, three were however received as late as 15 months after the survey.” | [33] |
| 2016 |  | Bites  Infestation | “Although his bedroom is sealed with silicone caulking, he was bitten numerous times in this room by adult *T. protracta*” (46-47).  “She was bitten numerous times by kissing bugs while sleeping […] In July, she finally searched the bedroom diligently and found one female and four 2nd instar nymphs of *T. protracta* between the mattress and box springs on her side of the bed” (47). | [34] |
| 2016 | San Diego  Los Angeles | Infestation | “*Triatoma protracta* specimens were actively collected from private residences in two study regions” (3).  “Hence, to augment the triatomine sample size from this region, we enlisted the help of property owners to collect bugs found in their homes” (4).  “In addition, we opportunistically obtained specimens from public health employees in southern California, who often received bugs from concerned citizens, especially if the bug had bitten someone within the home” (4).  “The five remaining bugs were adults obtained either light traps or within a resident’s home” (8).  “With the exception of one specimen from San Diego, these bugs represented a range of locations within the Greater Los Angeles Area: Agoura Hills, Altadena, Los Angeles, Northridge, Oak Hills, Santa Clarita, Simi Valley, Tarzana, and Thousand Oaks. Of the 11 bugs for which address were provided, area visualization via GoogleEarth revealed that the homes primarily abutted natural canyon areas designated as parks or were within housing tracts interspersed with parcels of undeveloped land’” (9). | [35] |
| *References not included in the main document are included below. | | | | |

1. Barber HG. A new genus and species of the subfamily triatominae (Reduviidae: Hemiptere). Proc Ent Soc Wash. 1938;40(4): 104-105.

2. Wood SF. Reactions of man to the feeding of reduviid bugs. J Parasitol. 1942a;28(1): 43-49.

3. Wood SF. Observations on vectors of Chagas’ disease in the United States. I. California. Bull So Calif Acad Sci. 1942c;41: 61-69.

4. Anonymous. Kissing bug is bad. California Farmer. 1950;193: 149.

5. Wood SF. Allergic sensitivity to the saliva of the Western cone-nosed bug. Bull So Calif Acad Sci. 1950a;49(2): 71-74.

6. Wood SF. The distribution of California Insect Vectors Harboring Trypanosoma cruzi Chagas. Bull So Calif Acad Sci. 1950b;49(3): 98-100.

7. Wood SF. Bug Annoyance in the Sierra Nevada Foothills of California. Bull So Calif Acad Sci. 1951a;19(3): 98-100.

8. Augustson GF, Wood SF. Notes on Californian mammals ecto-Parasites from the Sierra Nevada Hills of Madera County. Bull So Calif Acad Sci. 1953;52: 46-56.

9. Wood SF. Conenose bug annoyance and *Trypanosoma cruzi* Chagas in Griffith Park, Los Angeles. Bull So Calif Acad Sci. 1953c;52(3): 105-109.

10. Mehringer PJ, Wood SF. A resampling of wood rat houses and human habitations in Griffith Park, Los Angeles, for *Triatoma protracta* and *Trypansoma cruzi*. Bull S Calif Acad Sci. 1958;52: 46-56.

11. Wood SF. Conenose bug annoyance in Griffith Park in 1958. Bull So Calif Acad Sci. 1960b;59(1): 50-52.

12. Mehringer JP, Wood SF, Anderson RA. Conenose bug (Triatoma) annoyance and *Trypanosoma* cruzi in Griffith Park in 1960. Bull So Calif Acad Sci. 1961;60(3): 190-192.

13. Walsh JD, Jones JP. Public health significance of the cone-nosed bug, *Triatoma protracta* (Uhler), in the Sierra Nevada foothills of California. Calif Vector Views. 1962;9: 33-38.

14. Nichols N, Green TW. Allergic reactions to "kissing bug" bites. Calif Med. 1963;98: 267-268.

15. Scott K. Ken Scott catches the "kissing bug". PCO News. 1963;23: 17.

16. Swezey R. Kissing bug bite in Los Angeles. Arch Intern. 1963;112: 977-980.

17. Wood SF, Wood FD. Nocturnal aggregation and invasion of homes in southern California by insect vectors of Chagas’ disease. J Econ Entomol. 1964;57: 775-776.

18. Wood, S. D. and F. D. Wood. "New locations for Chagas’ trypanosome in California." Bull So Calif Acad Sci. 1964b;63(1): 104-111.

19. Wood SF, Anderson RC. Conenose bugs triatoma) visit unoccupied boy's camp in Los Angeles. J Med Entolmol. 1965;1: 347-348.

20. Sjogren RD, Ryckman RE. Epizootiology of *Trypanosoma cruzi* in southwestern North America. Part VIII: Nocturnal flights of Triatoma protracta (Uhler) as indicated by collections at black light traps (Hemiptera: Reduviidae: Triatominae). J Med Entomol. 1966;3: 81-92.

21. Wood SF, Wood FD. Ecological relationships of *Triatoma p. protracta* (Uhler) in Griffith Park, Los Angeles, California. Pac Insects. 1967;9: 537-550.

22. Nichols N. Data on bug sent to state Merced Sun-Star. 1975. California, Merced.

23. Wood SF. Additional notes on Chagas’ trypanosome in California and Arizona. Pan-Pac. Entomol. 1975;51(2): 167-168.

24. Wood SF. Reaction of man to the feeding of Triatoma protracta (Hemiptera: Reduviidae). National Pest Control Operator News. 1975a;35: 19-21.

25. Wood SF. Additional notes on the Western Conenose, Triatoma p. protracta (Insecta: Hemiptera: Reduviidae), as a carrier of Chagas' Trypanosome. Bull So Calif Acad Sci. 1978;77(3): 130-132.

26. Marshall N, Street D. Allergy to Triatoma protracta (Heteroptera: Reduviidae) Part I. J Med Entomol. 1982;19: 248-252.

27. Rohr AS, Marshall NA, Saxon A. Successful immunotherapy for Triatoma protracta-induced anaphylaxis. J Allergy Clin Immunol. 1984;73: 369-375.

28. Navin TR, Roberto RR, Juranek DD, Limpakarnjanarat K, Mortenson EW, Clover JR, et al. Human and sylvatic *Trypanosoma cruzi* infection in California. AJPH. 1985;75: 366-369.

29. Lane RS, Moss R, Hsu YP, Wei T, Mesirow ML, Kuo MM. Anti-arthropod saliva antibodies among residents of a community at high risk for Lyme disease in California. Am J Trop Med Hyg. 1999;61(5): 850-859.

30. Hwang WS, Guanyang Z, Maslov D, Weirauch C. Short report: infection rates of *Triatoma protracta* (Uhler) with *Trypanosoma cruzi* in Southern California and molecular identification of trypanosomes. Am J Trop Med Hyg. 2010;83: 1020–1022.

31. Klotz JH, Dorn PL, Logan JL, Stevens L, Pinnas JL, Schmidt JO, et al. Kissing bugs": potential disease vectors and cause of anaphylaxis. Clin Infec Dis. 2010;50: 1629-1634.

32. Stevens L, Dorn PL, Hobson J, de la Rua NM, Lucero DE, Klotz JH, et al. Vector blood meals and Chagas disease transmission potential, United States. Emerg Infect Dis. 2012;18:646-649.

33.Walter J, Fletcher E, Moussaoui R, Gandhi K, Weirauch C. Do Bites of Kissing Bugs Cause Unexplained Allergies? Results from a Survey in Triatomine-Exposed and Unexposed Areas in Southern California. Plos One. 2012;7(8): e44016. doi: 44010.41371/journal.pone.0044016.

34.Klotz SA, Mazda-Shirazi F, Boesen K, Beatty NL, Dorn PL, Smith S, et al. Kissing Bug (Triatoma spp.) Intrusion into Homes: Troublesome Bites and Domiciliation." Environ Health Insights. 2016;10: 45-49.

35.Shender L, Lewis M, Rejmanek D, Mazet J. Molecular diversity of *Trypanosoma cruzi* detected in the vector *Triatoma protracta* from California, USA. PLoS Negl Trop Dis. 2016a;10: e0004291.
